# Quantitative Assessment of the Sensitivity of Various Commercial Reverse Transcriptases Based on Armored HIV RNA

**DOI:** 10.1371/journal.pone.0013931

**Published:** 2010-11-10

**Authors:** John B. A. Okello, Linda Rodriguez, Debi Poinar, Kirsten Bos, Andrew L. Okwi, Gabriel S. Bimenya, Nelson K. Sewankambo, Kenneth R. Henry, Melanie Kuch, Hendrik N. Poinar

**Affiliations:** 1 Department of Anthropology, McMaster Ancient DNA Centre, McMaster University, Hamilton, Ontario, Canada; 2 Department of Pathology, School of Biomedical Sciences, College of Health Sciences, Makerere University, Kampala, Uganda; 3 Office of the Principal, College of Health Sciences, Makerere University, Kampala, Uganda; 4 Division of Infectious Diseases, Case Western Reserve University, Cleveland, Ohio, United States of America; 5 Michael DeGroote Institute for Infectious Disease Research, McMaster University, Hamilton, Ontario, Canada; 6 Department of Pathology and Molecular Medicine, McMaster University, Hamilton, Ontario, Canada; St George's University of London, United Kingdom

## Abstract

**Background:**

The *in-vitro* reverse transcription of RNA to its complementary DNA, catalyzed by the enzyme reverse transcriptase, is the most fundamental step in the quantitative RNA detection in genomic studies. As such, this step should be as analytically sensitive, efficient and reproducible as possible, especially when dealing with degraded or low copy RNA samples. While there are many reverse transcriptases in the market, all claiming to be highly sensitive, there is need for a systematic independent comparison of their applicability in quantification of rare RNA transcripts or low copy RNA, such as those obtained from archival tissues.

**Methodology/Principal Findings:**

We performed RT-qPCR to assess the sensitivity and reproducibility of 11 commercially available reverse transcriptases in cDNA synthesis from low copy number RNA levels. As target RNA, we used a serially known number of Armored HIV RNA molecules, and observed that 9 enzymes we tested were consistently sensitive to ∼1,000 copies, seven of which were sensitive to ∼100 copies, while only 5 were sensitive to ∼10 RNA template copies across all replicates tested. Despite their demonstrated sensitivity, these five best performing enzymes (Accuscript, HIV-RT, M-MLV, Superscript III and Thermoscript) showed considerable variation in their reproducibility as well as their overall amplification efficiency. Accuscript and Superscript III were the most sensitive and consistent within runs, with Accuscript and Superscript II ranking as the most reproducible enzymes between assays.

**Conclusions/Significance:**

We therefore recommend the use of Accuscript or Superscript III when dealing with low copy number RNA levels, and suggest purification of the RT reactions prior to downstream applications (eg qPCR) to augment detection. Although the results presented in this study were based on a viral RNA surrogate, and applied to nucleic acid lysates derived from archival formalin-fixed paraffin embedded tissue, their relative performance on RNA obtained from other tissue types may vary, and needs future evaluation.

## Introduction

Quantitative polymerase chain reaction (qPCR) is today one of the most accurate methods used for the quantification of nucleic acids in genomic studies [1], biotechnology and forensics [2], and was recently utilized to detect archival retroviruses [3]. For RNA detection [4], the most fundamental step is the *in-vitro* reverse transcription (RT) of RNA to its complementary DNA (cDNA), catalyzed by the enzyme reverse transcriptase [5], [6], [7]. Many factors such as the quality of the RNA, the presence of inhibitors co-extracted with the RNA, as well as the RT efficiency are known to affect the total yield of cDNA [8], [9].

While the success of obtaining cDNA from samples with low copy number RNA levels will depend mainly on the reverse transcription step [8], [10], [11], it is known that certain reaction components have inhibitory effects on downstream applications, especially qPCR [5], [12], [13], [14], [15], [16]. In fact, some studies have shown the main inhibitory substance is the reverse transcriptase enzyme itself [12], [13]. As such, the enzyme needs to be removed or denatured directly after the RT reaction is complete to reduce its inhibitory and/or nuclease activities. This can be achieved via a heat or an alternative inactivation step which has been incorporated in almost all commercially available reverse transcriptase kits/systems. However, with the increasing number of heat-resistant RT enzymes [9], and the fact that many RT enzymes retain their inhibitory properties even post denaturation [15], there is need for alternative strategies to overcome this limitation. Dilutions of RT products are often used to minimize post-RT inhibitory effects [5], [10], [12], [13], [17], [18], although this has the negative consequence of reducing quantification precision, which is especially problematic when dealing with very low copy templates, such as those obtained from archival tissues.

Most biological applications utilizing RNA, such as gene expression assays, generally rely on relative amounts of RNA as opposed to discrete copy numbers, hence the absolute amount of total RNA in a sample is of little importance [19]. In addition, sensitivities with low templates are not generally of concern for such assays, as expression levels are expected to be high. These factors, however, are of paramount importance when dealing with samples containing very low amounts of nucleic acid [16] or highly fragmented RNA such as viral RNA recovered from archival Formalin-Fixed Paraffin Embedded (FFPE) tissues [3]. For the latter application, it is advantageous to use the most sensitive RT enzyme available [20]. Given the increasing attempts by many researchers to quantitatively determine RNA levels from very low or highly degraded sources using commercially available reverse transcriptases, it is surprising that their analytical sensitivity, efficiency, and reproducibility are not extensively explored.

Most of the commercially available reverse transcriptases are derived from Avian Myelomatosis Virus (AMV), Murine Moloney Leukemia Virus (MMLV) and/or the Human Immunodeficiency Virus (HIV). While it is claimed that these enzymes produce high yields of cDNA during reverse transcription [21], some notable studies have compared their relative performances [10], [21], [22], [23], [24], and to our knowledge, only two have attempted to evaluate the suitability of a few of these common RT enzymes in the reverse transcription of low copy number RNA [10], [12]. Using AMV-RT, Chandler et al. [12] assessed inhibition of qPCR at low template concentrations (2 fg – 2 pg), while Levesque-Sergerie et al. [10] tested detection limits of five commercial reverse transcriptases on a template range of 10–2,000 ng. Both of the above mentioned studies were largely qualitative. Similar to the previous studies, Sieber et al. [24] experimented on the sensitivity of 9 RT enzymes, however detection limits were not tested and their results were based on RNA obtained from different tissues, potentially prone to variations. Two other studies have documented high sensitivity of RT-qPCR assays aimed at detecting HIV-1, with a limit of 5 copies per ‘reaction’ and 1 copy/ml respectively [25], [26]. While these are interesting results, their study were each based on only one RT enzyme, among the many commercial ones available which we tested herein.

The purpose and scope of this study therefore was to compare the suitability of 11 commercially available reverse transcriptases with an RT-qPCR assay for the sensitive detection of very low template amounts. Using cDNA produced from increasing quantities of Armored HIV RNA surrogate, we compared the analytical sensitivity (detection limits), linearity of product amplification, intra- and inter-assay variability, and qPCR amplification efficiency of all of these RT-systems. We also tested the applicability of our preferred RT enzyme in quantifying viral RNA from nucleic acid extracts derived from a formalin fixed paraffin embedded tissue. Since sample variations or differences in sample preparations may influence any observed difference in enzyme efficiency [27], we used a well tested primer pair and viral RNA standard, the Armored HIV RNA [28], [29], thereby eliminating potential variation due to template sources. Overall, this evaluation should be fundamental in guiding researchers striving to quantitatively detect low copy number RNA or rare transcripts in genomic studies.

## Materials and Methods

### Ethics Statement

This study uses nucleic acid lysates from an archival human visceral FFPE tissue in some of its assays. The approval for the use of this tissue was obtained from the Institutional Review Boards (IRB) of both the College of Health Sciences at Makerere University and the Faculty of Health Sciences at McMaster University. Since the tissue in question was anonymous and post-mortal, no informed consent was necessary, save for the above IRB approval of its use in this study.

### General overview of the study

The archival FFPE tissue was extracted as previously reported [30], and used to examine the suitability of the different RT enzymes in successful amplification of nucleic acid from a typical archival tissue lysate, but with known quantity. Prior to all experimental setup, all glass, tubes, plastic wares and working surfaces were bleached and treated with RNAse inhibitor, RNase Zap (Ambion Inc, Austin, TX, USA), and all solutions were prepared with diethylpyrocarbonate-treated water.

In quantitative assessment of the sensitivity of various commercially available reverse transcriptases, the absolute copy numbers of the target RNA molecules were calculated using appropriate standard curves [31], [32], from duplicate dilution series of the Armored RNA Quant HIV standard (Asuragen, Inc. Austin, TX, USA) during the reverse transcription stage [19], [33]. Standard curves provide a simple, rapid and reproducible indication of efficiency, analytical sensitivity, and the variability of each assay [32]. The number of amplification cycles needed to reach the crossing point (Cq) was used to determine the starting template amount in reactions where we assumed unknown template amount. The Armored RNA Quant HIV (hereafter referred to as Armored HIV RNA), is a viral RNA surrogate normally used in extraction control, transcription or PCR-detection. It consists of a 456 bp GAG region sequence derived from HIV-1 B (HXB), encapsulated within an MS2 bacteriophage particle that renders it resistant to RNase digestion [29], [34], [35].

### RT setup and reaction

First-strand cDNA was synthesized from each serially diluted Armored HIV RNA using all the 11 reverse transcriptase enzymes evaluated in this study (Table 1). To avoid potential template variation between assays, we used the same vial of Armored HIV RNA, heated to 70°C for 3 min to release the RNA from its bacteriophage-like complexes before cDNA synthesis. Fresh RNA dilutions, ranging from 5–500 copies/µl were used for each assay to avoid potential variation due to freeze-thaw cycles, which could influence the quality of RNA. All RT reactions were prepared using a single mastermix, strictly following the manufacturer's protocol (see supplementary Table S1 for detailed summary of each protocol). In summary, each reaction was carried out in 20 µl reaction volume using the HIV gene specific primer (GSP) SK431 [34]. Each duplicate reaction included the manufacturer recommended amounts of GSP primer, dNTP mix, RT buffer, DTT, RNase Inhibitor (except SML), Armored HIV RNA template and reverse transcriptase enzyme, respectively.

**Table 1:** Commercial Reverse Transcriptases compared in this study.

RTase	Brand Name	Acronym	RT Enzyme	Cat. No.	Company
1	AccuScript® High Fidelity 1st Strand cDNA Synthesis Kit	ACC	MMLV, H^−^	200820	Stratagene-Agilent Technologies, La Jolla, CA USA
2	AMV Reverse Transcriptase	AMV	AMV	M5101**	Promega Corporation, Madison, WI USA
3	Recombinant HIV Reverse Transcriptase	HIV	HIV	AM2045**	Ambion-Life Technologies Corp, Carlsbad, CA USA
4	ImProm-II® Reverse Transcription System	IP2	proprietary source	A3800**	Promega Corporation, Madison, WI USA
5	M-MLV Reverse Transcriptase	MML	MMLV, ^↓^H^−^	M170A**	Promega Corporation, Madison, WI USA
6	Protoscript® First Strand cDNA Synthesis Kit	PRS	AMV	E6500S*	New England Biolabs, Ipswich, MA, USA
7	Sensiscript® RT Kit	SES	proprietary source	205211	QIAGEN GmbH, Hilden, Germany
8	Smart MMLV Reverse Transcriptase	SML	MMLV	639523	CloneTech Laboratories Inc, Mountain View, CA USA
9	Superscript II® Reverse Transcriptase	SS2	MMLV, ^Ω^H^−^	18064-014	Invitrogen-Life Technologies Corp, Carlsbad, CA USA
10	Superscript III® Reverse Transcriptase	SS3	MMLV, ^Ω^H^−^	18080-044	Invitrogen-Life Technologies Corp, Carlsbad, CA USA
11	ThermoScript™ RT-PCR System for First-Strand cDNA Synthesis	TSR	AMV, ^Ω^H^−^	11146-024	Invitrogen-Life Technologies Corp, Carlsbad, CA USA

*Supplied to us at 25% discount.

**Supplied to us free of charge

RT = Reverse Transcriptase; HIV = Human Immunodeficiency Virus; AMV = Avian Myeloblastosis Virus; MMLV = Moloney Murine Leukemia Virus Reverse Transcriptase; ^Ω^H^−^ = Point mutation that eliminates RNase H^−^ activity, and ^↓^H^−^ = reduced RNase H^−^ activity.

### Quantitative PCR assay

The RT products were amplified using primers SK462 and SK431 to generate a 142 base pair amplicon [34]. The qPCR reactions were performed using the MxP - Mx3000P Real Time PCR System (Stratagene -Agilent Technologies, La Jolla, CA USA). Each 20 µl reaction contained 1× PCR Buffer II, 2.5 mM MgCl_2_, 1.0 µg/µl bovine serum albumin (BSA), 250 µM of each deoxynucleoside triphosphate (dNTP), 250 nM of each primer, 0.167× SYBRGreen I, 0.05 U/µl AmpliTaq Gold DNA polymerase, and 2 µl of cDNA (or water for nontemplate controls). The amplification profile consisted of 95°C for 7 min, 45 cycles of 95°C denaturation for 30 s, annealing at 65°C for 30 s, extension at 72°C for 30 s, and a final extension at 72°C for 10 min. SYBR Green I based fluorescence data were acquired during each annealing phase of the reaction, while a melting curve was generated after the 45^th^ amplification cycle to check amplification specificity of RT-qPCR. All our analyses involved the manual removal of background fluorescence using the MxPro - MX3000P v4.10 (Stratagene) after each run.

In determining the level of RT-qPCR amplified products, the quantification cycle was assumed to be proportional to the starting Armored HIV RNA molecules in each dilution assayed. The analytical sensitivity and efficiency of each reverse transcriptase system was determined by amplifying cDNA derived from serial dilutions of the above armoured HIV surrogate, using the same mastermix in each qPCR assay. Another set of assays was done independently on a different day to test for reproducibility across runs.

### Assessing repeatability and reproducibility

The repeatability of the tested RT enzymes in reverse transcription was estimated as the standard deviation (sd) of intra-assay quantification cycle (Cq) variance, while the reproducibility (inter-assay variability) was measured as the coefficient of variation (cv) of an estimated copy number between different runs. The latter were assayed based on ACC (details in Table 1), preliminarily the most sensitive RT enzyme (its standard curve was then used to estimate quantities in other enzymes). This test was conducted using only the highest standard copy (1,000 copies per reaction), and the variances were calculated both within and between runs. Overall, the mean Cq and corresponding sd between replicates within the same run, and percentage cv of amplified copies between runs were calculated to assess reproducibility of each RT enzyme tested in accordance with the Minimum Information for Publication of Quantitative Real-Time PCR Experiments (MIQE) guidelines [32].

### Establishing the standard curves and correlation coefficients

The standard curve was generated by performing serial dilutions of the armored HIV RNA and assaying each dilution twice per RT enzyme, while using a non-template control in each assay to check for contamination. Since all reverse transcription reactions were carried out in 20 µl reaction volumes, the template RNA amount ranged from 10, 100 and 1,000 copies per reaction (≈1–100 fg/µl). To determine linearity in cDNA synthesis of the different assays, we amplified the three aforementioned dilutions, plotted the Cq values against the amplified copies, and calculated the linear regression of the curve as well as the correlation coefficient (Rsq). The quality of the standard curve generated from each RT reaction was judged via the slope and its Rsq, as well as the amplification efficiency using the software Mx3000P. In theory, the slope should be −3.3, representing a theoretical doubling stemming from a 1∶10 dilution series. So, an RT-qPCR yielding a slope lower than −3.3 would wrongly indicate its efficiency is greater than 100% and hence each quantification cycle would be generating more than twice the amount of cDNA copies. If this is observed, it would be attributable to the RT enzyme system itself, since the primers and qPCR reagents were consistent across all RT enzymes tested.

### Assessing performance of RT enzymes with FFPE tissue

For a comparison of how the different RT enzyme systems would synthesize cDNA from a typical archival FFPE tissue extract, we spiked all nucleic acid extracts from a typical FFPE tissue of 1995 [30] with a known amount of RNA standard and compared their cDNA yields to those obtained from the same amount of RNA standard assayed under identical conditions, though in the absence of extract. The Cq measured from the spiked reaction was compared with that of the control reaction, with the assumption that an RT enzyme performing well in the presence of a typical FFPE tissue extract should show similar or increased Cq relative to that in the un-spiked reference standard. This assay enabled testing/comparing the relative performance of the RT enzymes in reverse transcribing RNA from a typical FFPE tissue extract with similar RNA quantity, but also indirectly shows how potential co-purified inhibitors in the FFPE tissue would affect RT-qPCR accuracy.

### Inhibition and post-RT cleanup method

To assess the potential benefits of post RT purification on qPCR inhibition, we chose a single enzyme (ACC) and performed a reaction in which we spiked the reactant cDNA product into a qPCR amplification with a known standard, 1×10^3^ copies/µl of a purified cloned-PCR product (cytochrome b) of mammoth [36]. We then compared the Cq obtained from the spiked amplification with that of a control reaction, with the expectation that any observed delay (shift) in the Cq relative to that of the amplified control would be an indication of inhibition [37]. We then tested two different purification methods to choose the most suitable one for post-RT cleanups; i) PCI-Microcon, the phenol-chloroform-isoamyl alcohol purification with subsequent Microcon YM-30 Centrifugal Filter Unit concentration (Millipore, Temecula CA, USA), and ii) MiniElute, the MinElute PCR Purification Kit (QIAGEN, Hilden, Germany), and compared them with both straight and diluted RT products. Briefly, the PCI–Microcon involved bringing the RT aliquots up to 100 µl with 0.1xTE buffer (pH 7.5), and 50 µl of 25∶24∶1 phenol/chloroform/isoamyl alcohol was added to each subsample, mixed gently by vortexing before spinning at 13,200 rpm in a microcentriguge for 2 min. The aqueous phase from above was re-extracted with 50 µl of chloroform before concentration using the Microcon YM-30 according to the manufacturer's instructions. The MiniElute protocol on the other hand was followed as described by the manufacturer (QIAGEN). In all cases, the final eluates were made to the same volume as the initial starting RT product volume so that no later adjustments in volumes would be necessary.

### Data analysis

We determined the correlation coefficient (Rsq), the slope and hence efficiency of each assay using the Mxp-Mx3000P software, employing the same threshold setting within each run. Other data analyses, including assay precision (measured from the coefficient of variation), standard deviation and statistical significance of observed differences in results were calculated using the Data Analysis Tools in the Microsoft Office Excel (Microsoft Corp, USA), and R version 2.11 [38].

## Results

The serially diluted HIV RNA used in this study ranged from 500 copies/µl (56.40 fg/µl) to 5 copies/µl (0.564 fg/µl). Further dilutions were not attempted as it would be below a reliable PCR detection limit. Typically, the theoretical analytical sensitivity of a given PCR with certainty is approximately 3 copies [39], assuming a Poisson distribution, and a 95% chance that at least 1 copy is detected in the PCR.

### Repeatability, reproducibility and sensitivity

Generally, melt curve analysis confirmed the majority yielded products of expected size, but some of them had multiple peaks that suggested multiple products (Figure S1) or lacked any amplified product (failed reactions), many of the latter stemmed from the lower RNA template dilutions. The RT enzymes that produced erroneous efficiencies due to non-specificity (eg SML) or failed altogether at the low copy end were excluded from further analysis that required all dilution points. In brief, six RT enzymes amplified the low RNA copy number standards (five of them consistently across all replicates tested), while most enzymes amplified the high copy number standards both within and between assays.

**Figure 1:**
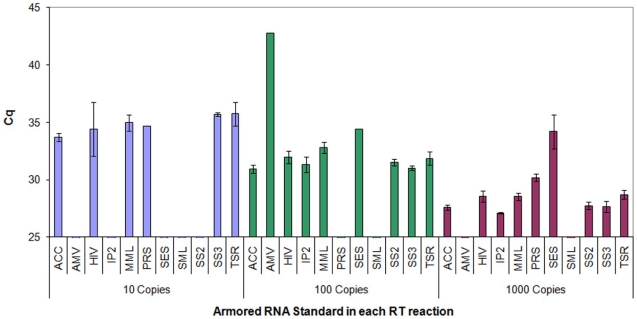
Average quantification cycles (and their standard deviations, represented as error bars) from 10, 100, 1000 copies of Armored HIV RNA assayed using all 11 reverse transcriptases compared in this study. The coloring corresponds to the copies of Armored RNA in each reaction. Only Cq points above 25 cycles are shown to emasize the differences among the enzymes assessed.

**Figure 2:**
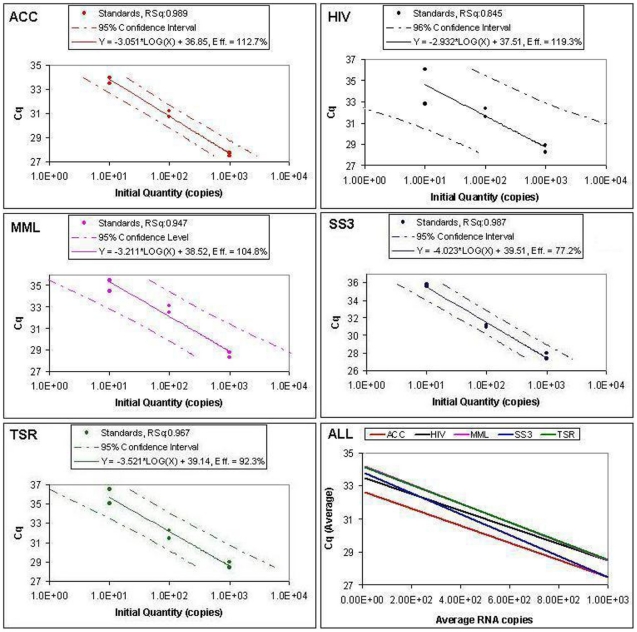
Standard curves for the five most reproducible reverse transcriptases that amplified all Armored HIV RNA replicates tested in this study. The standard curves (solid lines) and their associated 95% confidence intervals (dashed lines) were generated by the Mxp-MX3000P software by plotting quantification cycles (Cq) against RNA molecules (10–1,000 copies) amplified in duplicates. Only Cq points above 25 are shown in the graphs to emphasize phthe differences among the enzymes assessed. Correlation coefficients (Rsq) and amplification efficiencies (Eff.) as well as the linear equation of each curve are shown. The tightness of the 95% confidence limit shows quantification accuracy given an input RNA template amount.

Only a few did not work consistently across runs (Figure 1), leaving 7 of the 11 amplifying all replicates at the high end (Figure S2). Ultimately, we were left with only 5 RT enzyme systems (ACC, HIV, SS3, MML and TSR), where we could estimate and compare amplification Rsq as well as efficiency (Figure S3), with their standard curves varying in linearity and efficiency (Figure 2), emphasizing their variation in analytical sensitivity, especially at low copy number RNA levels.

The Cqs of each standard dilution point across replicates were generally similar although SS3 and ACC RT enzymes were the most consistent in their amplification of low copy number templates (Figure 1). The overall technical variability, calculated as average percentage coefficient of variation showed SES (cv = 4.27) and HIV (cv = 3.41) as the two most variable RT enzymes, while SS3 (cv = 0.89) and ACC (cv = 1.00) as the least variable and thus the most reproducible RT enzymes. In terms of technical reproducibly at the lowest copy numbers, the best two RT enzymes were SS3 (sd = 0.39%) and ACC (sd = 1.04%), while overall, ACC remained the most reproducible (sd = 1.44%), with the least reproducible enzyme being MML (sd = 137.63%); detailed results (see Table 2).

**Table 2:** Sensitivity as well as intra-assay and inter-assay reproducibility of the 11 reverse transcriptase enzymes assessed in this study.

RT	ACC	AMV	HIV	IP2	MML	PRS	SES	SML	SS2	SS3	TSR
***A: Sensitivity and reproducibility***											
**10**	33.69±0.35	NA	34.43±2.34	NA	34.97±0.71	34.68	NA	NA	NA	35.69±0.14	35.75±1.01
**100**	30.94±0.37	42.78	31.97±0.56	31.31±0.68	32.80±0.49	NA	34.42	NA	31.54±0.28	31.04±0.18	31.87±0.60
**1000**	27.59±0.21	NA	28.53±0.48	27.11±0.08	28.52±0.33	30.17±0.30	34.20±1.46	NA	27.76±0.32	27.63±0.47	28.69±0.38
***B: Intra-assay variability***											
**10**	1.04	NA	6.80	NA	2.03	NA	NA	NA	NA	0.39	2.83
**100**	1.20	NA	1.75	2.17	1.49	NA	NA	NA	0.89	0.58	1.88
**1000**	0.76	0.31	1.68	0.30	1.16	0.99	4.27	NA	1.15	1.70	1.32
**Average**	1.00	0.31	3.41	1.23	1.56	0.99	4.27	NA	1.02	0.89	2.01
***C: Inter-assay variability***											
1000	1.44	NA	64.75	7.43	137.63	NA	NA	NA	4.99	21.31	63.68

sd = standard deviation, cv = coefficient of variation. A) Sensitivity and reproducibility (mean Cq±sd, n = 2) within a run; B) Intra-assay variability [% cv = (sd/mean Cq)x100] based on replicate runs; C) Inter-run variability of cDNA quantification derived from 1,000 copies of Armored HIV RNA with ACC reverse transcriptase providing the calibration curve. NA denotes failed amplifications or values excluded due to non-specific products, while Cq values with no sd amplified only once.

### Standard curves, correlation coefficients (Rsq) and efficiency

The standard curve method, commonly used to estimate target DNA amounts from unknown samples based on a serially diluted standard, requires the amplification efficiency in the samples to be the same as those in the standards used [19]. The 5 enzymes exhibited clear linear relationships among the dilution points and Cq values obtained from amplification of Armored HIV RNA, with each yielding an Rsq of ≥0.95, except for the HIV RT enzyme (0.85). The standard curves for the five best enzymes as judged by consistency and efficiency are shown in Figure 2, with their 95% confidence intervals. Also included are the correlation coefficients (the linearity of each RT enzyme tested) as well as their efficiencies. The qPCR efficiencies calculated from the standard curves were generally high, ranging from 119.3 to 77.2%, suggesting reverse transcription was similarly efficient for these enzymes.

### Performance of commercial RTs in the presence of FFPE extract

The RT enzymes varied considerably in their ability to reverse transcribe Armored HIV RNA spiked with a typical FFPE tissue extract, showing seven of the enzymes to consistently amplify 1,000 copies of Armored RNA tested (Figure S2). Overall, ACC, TSR and SS3 exhibited the most superior capability to synthesize cDNA from FFPE tissue lysates as suggested by their very small Cq (sd) and consistent amplification across all replicates tested. The other RT systems (not indicated), completely failed, were irreproducible or displayed high intra-assay variability in relation to the un-spiked standard (Figure S4).

### Post-RT cleanup minimizes RT inhibitory effect

**Figure 3:**
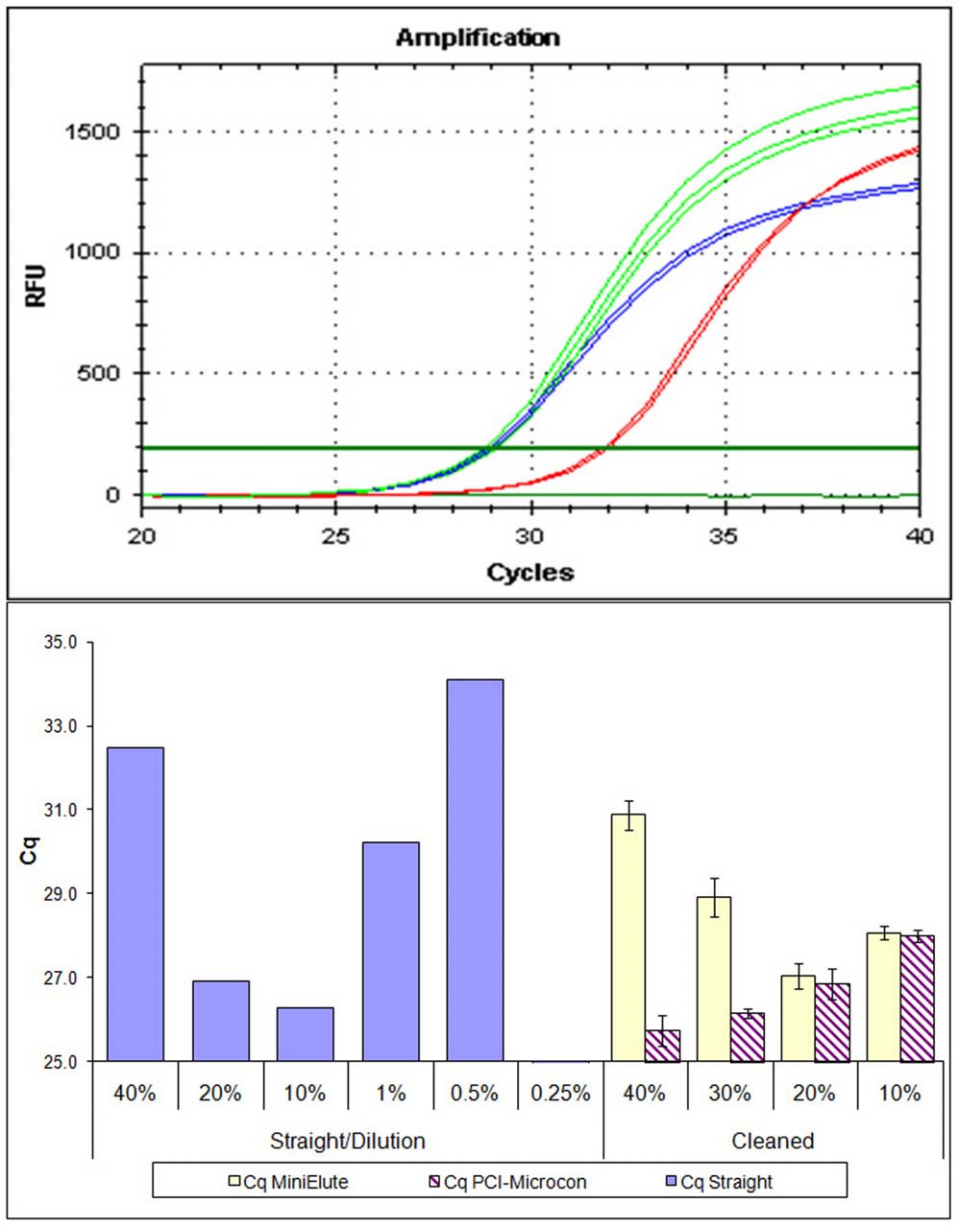
Comparison of amplification success measured by quantification cycle (Cq) of the straight cDNA with post-RT processed cDNA. Post-RT processing was by means of dilutions, phenol-chloroform-isoamylalcohol-microcon and MiniElute purification of ACC generated RT products, the latter two in tripcates. A) Typical amplifications profile of a DNA standard (green), compared to ones spiked with RT unpurified (straight; in red) and PCI-Microcon YM-30 cleaned products (blue). The shift in Cq when the amplification reaction included straight RT products demonstrates the inhibitory effects of RTs. B), comparison of the strategies used to reduce inhibitory effects of RT, with blue bars showing dilutions (as percentage reduction of straight template) and increasing amounts of post-RT cleaned products (depicted as purple stripes, superimposed on yellow bars). Only Cq points above 25 were shown in the graphs to emphasize the differences revealed. Straight/Dilution = Straight Armored RNA templates range from 0.25–40%, and PCI-Microcon = phenol-chloroform microcon cleaned Armored RNA products.

While the impact of inhibitory components of RT reactions on known standards have been previously evaluated [9], no study to our knowledge has systematically evaluated such a large number of enzymes as tested here. The results of our inhibition tests showed a clear inhibitory effect of the RT reagents on qPCR as demonstrated by a Cq shift in the quantification cycle relative to the PCI-Microcon cleaned cDNA (Figure 3), but not statistically significant (Wilcoxon–Mann–Whitney test, *P* = 0.1). However, of the two purification methods tested, the PCI–Microcon yielded significantly more number of template molecules than the MiniElute purified ones (Wilcoxon–Mann–Whitney test, *P*<0.005). The two methods, however, did not significantly differ when ≤20% of the RT-reactions were used in the qPCR setup. Therefore, using more than 20% RT products in a qPCR reaction certainly reduces amplification success, as demonstrated by a delayed Cq shift, and thus if cDNA syntheses are to be used for downstream PCRs detection of low copy templates, it would be wise to purify RT reaction products before further assay.

## Discussion

In order to find the most suitable RT enzyme for amplifying low copy RNA, a total of 11 commercial reverse transriptases derived from either Moloney Murine Leukemia Virus (ACC, MML, SML, SS2 & SS3), Avian Myeloblastosis Virus (AMV, PRS & TSR), Human Immunodeficiency Virus (HIV), or other proprietary sources (IP2 and SES) were compared (Table 1). Four of these enzymes were engineered with RNAse H minus point mutations to render them non-degrading to RNA, while one had RNase H activity substantially reduced, in addition to other characteristics and optimizations (see Tables 1 & S1 for more information). The suitability of the RT enzymes in reverse transcribing RNA from FFPE tissue extracts was also assessed. All results were based upon the quantitative amplification of a known number of armored HIV RNA copies using a widely accepted standard curve method [19]. To the best of our knowledge, our study represents the first to analyse the lowest quantitatively known copy number RNA templates, a level more likely to be found in RNA extracts from archival formalin fixed tissues. The results presented in this study were based on a viral RNA surrogate, and applied to nucleic acid lysates derived from archival FFPE tissue based on gene-specific primers. As such, their relative performance may vary if RNA obtained from other tissue types or random primers are used, however evaluation of this is beyond the scope of the present study.

### Reproducibility and efficiency of RT reactions

While the results presented here largely agree with the Invitrogen (Life Technologies) assertion that their SuperScript® family of reverse transcriptases delivers reliable and consistent results, we hereby show that other enzymes perform equally well and consistently, especially at low template amounts. From the 11 RT enzymes studied, five enzymes (ACC, HIV, SS3, MML and TSR) produced reproducible results across all dilution points tested. The other RT enzymes all exhibited nonspecific products in the melt curves (Supplemental Figure S1), with efficiencies sometimes greater than 100%, and hence were excluded, leaving only five best performing enzymes (Figure 2) for further analysis. Overall, these five reverse transcriptases either had no or comparatively reduced RNase H activity, suggesting that mutant RNase-H- RT enzymes might out perform their generic counterparts. Using the ACC based calibration curve, we observed the most reproducible yield from SS2 and SS3 enzymes. The amplified 1,000 RNA copies were slightly overestimated for the IP2 enzyme (Figure S2), and the lowest inter-assay variation was observed in ACC, followed by SS3, while HIV RT had the highest variation (Figure 2 and Table 2).

### Analytical sensitivity and efficiency of reverse transcriptases

While the majority of the total eleven enzymes failed to consistently amplify across the range tested (Table 2), the five which performed well at the lowest template end tested (Figure 1), are more suitable for studies aiming at detecting low amounts of RNA templates. In particular, ACC and SS3 demonstrated the most analytical sensitivity and reproducible enzymes across all dilution points (Table 2, Figure 2), with overall superiority at low amounts of viral RNA tested. There are many reasons for failed amplifications at low copies, as has been previously noted elsewhere [8], [40], [41]. Inter-assay reproducibility of the different enzymes based strictly on the highest template amounts was greatest in ACC and SS2 (Figure S2), with their percentage coefficients of variation at 1.44 and 4.99 respectively (see Table 2 for more details). Overall, our results corroborate a previous study that assessed the efficiency and sensitivity of different commercial reverse transriptases, where reverse transcription at high template numbers was efficient for all RTs tested, but low template amounts could not be detected with certain RT systems [10].

Two previous studies have reported high sensitivity of RT-qPCR assays for the detection HIV-1, with their limits at 5 copies per reaction and 1 copy/ml respectively [25], [26]. These two studies did not compare different RT systems but rather focused on MultiScribe and SuperScript II RT enzymes respectively. While there are a few other commercially available reverse transcriptases on the market, and potentially in individual laboratories, our study represents one of the most comprehensive evaluations of the major commercial RT enzymes available on the market today. Contrary to a study by Levesque-Sergerie et al. [10], which found most RT enzymes tested were not able to reverse transcribe below 2,500 RNA copies (except for Superscript II), we observed that 9 of the 11 RT enzymes tested in this study could detect 1,000 Armored HIV RNA copies, across all replicates assayed, while seven of them were sensitive to 100 copies, and only five of them consistently amplified over the entire range tested (10–1,000 RNA copies, see Table 2 and, Figures 1 & 2). Interestingly, SS2 or SES were not among these, though they were the two best enzymes reported by Levesque-Sergerie et al. [10]. The lack of sensitivity of some RT systems at low template RNA amounts could be due to either PCR inhibitors or preferential primer-dimer formation [10], [13]. Primer-dimer formation can be exacerbated by limited template or extended amplification cycles beyond the linear phase of PCR [42]. These factors likely also lead to the occurrence of artificially inflated PCR amplification efficiencies over 100% [15]. Due to lack of sensitivity [43], there is clearly a need for post-RT purification in order to remove the reverse transcriptase and the RT components prior to performing PCR [15], or the use of mutant Taq DNA polymerases that are resistant to these inhibitors [44]. Since cleaning up the reactions seems beneficial, we investigated post-RT cleaning methods for low template RT-products as discussed below.

### Post-RT cleaning methods appropriate for low template RT-qPCR

The cDNA synthesis and the successful use of its product in PCR amplification may be influenced by numerous factors, and this may depend on the type of reverse transcriptase enzyme used. Previous studies have suggested that reverse transcriptase enzymes inhibit PCR at low template concentrations [5], [12], [13], [14]. Two studies have documented simply using up to half of or all of the RT reaction (after heat-killing the RT) in the subsequent PCR reaction without any apparent problem with the sensitivity of the subsequent PCR step [25], [26]. This is in contrast to our paper, which suggests a strong inhibitory effect. This may be due to the less inhibitory effect of the reagents in the respective RT systems or PCR enzymes used after RT are inhibition resistant. While simply increasing template concentrations might be useful when dealing with modern and invasively obtained DNA sources, such a strategy would not work for low copy viral RNA from highly degraded archival FFPE tissues, especially if the same extracts are needed for multiple tests/targets.

The inhibition test we performed [36], [45] confirmed inhibitory effect of RT on qPCR, depicted by the straight (unpurified) RT products being substantially delayed in their qPCR Cq relative to the standard (Figure 3A). While RT dilution seems the easiest way to overcome this inhibition, as has been widely recommended to augment qPCR amplification success [5], [12], [13], [14], this strategy has the negative consequence of reducing the number of available template copies, and is thus not a suitable alternative when template molecules are expected to be minimal. Diluting the RT products before downstream application [5] likely yields inconsistent and non-reproducible detection [8], [11], owing to competition between RT molecules and DNA polymerase I in the PCR, thereby decreasing the reaction efficiency [15]. For this reason, we tested the efficacy of removing post-RT inhibition using two alternative cDNA methods; the PCI-Microcon and the MiniElute purifications.

Based on our results, it is clear there is a comparative advantage in cleaning the RT products as opposed to using them straight or diluted. Specifically, we observed that post-RT cleaning with the PCI-Microcon method enhances the qPCR success, yielding relatively more amplified products even at increased template amounts (Figure 3B). In addition to being a better method at limiting the RT inhibition, we hypothesize that this purification method might be better at releasing the RNA:DNA complexes and hence increasing cDNA availability for qPCR amplification. We also found that up to 40% of the PCI-Microcon purified RT product could be used in a qPCR without substantially inhibiting the reaction (Figure 3), whereas increasing template volume beyond 20% for MiniElute cleaned RT product met with increased Cq, suggesting interference with amplification. This could be due to competition of the RT molecules with DNA polymerase I in qPCR thereby decreasing the reaction efficiency [15]. Increasing template amount beyond 10% substantially delays Cq relative to the standard, suggesting increased inhibitory effect on qPCR at this stage. Although the reasons for the differences between the two methods are not clear, relatively more inhibitory properties still remains after MiniElute purifications as reported previously [28], [46].

### Conclusions

Our results suggest that, of the 11 reverse transcriptase enzymes subjected to our investigation, the Accuscript (ACC) and Superscript III (SS3) were the best performing enzymes in terms of reproducibility and sensitivity for low copy RNA levels. For those wishing to quantify low RNA template molecules, it is advisable to augment the detection via use of a PCI-Microcon purification step, and where necessary use up to 40% of the purified RT product in downstream applications, such as qPCR. This option is favored over simple dilution to minimize inhibition when template amounts are presumed to be minimal.

## Supporting Information

Table S1 Detailed reverse transcription steps, reaction conditions and ingredients for all the 11 commercial reverse transcriptases studied. The same colour coding at each stage show groups enzymes that were incubated together for the respective stage, the rest of the stages were all done in unison.
(0.04 MB XLS)

Figure S1 The qPCR profile of all the amplifications conducted in this study, showing clearly differentiated melt curves. Melt curve of the expected products peaked at ∼84°C, while those from the unspecific amplicons (erroneous amplifications) peaked at <82°C, the latter comprised largely of primer dimmers. Because of many number of amplifications combined in this figure, it was impossible to provide the legend to each curve.
(0.60 MB EPS)

Figure S2 Intra-run and inter-run variability of the 11 commercial reverse transcriptases (mean±sd, n = 2) based on amplification of cDNA produced from 1,000 copies of Armored HIV RNA. A) shows the results presented across runs in ascending order, while B are the means across runs of estimated cDNA copies produced between different assays. The comparisons were based on quantities estimated with ACC reverse transcriptase providing the calibration curve, for all other RT enzymes. AMV (Promega) failed in the second experiment and hence couldn't be compared, while we excluded SML (CloneTech) due to the non-specific products consistently obtained across the different runs.
(0.29 MB EPS)

Figure S3 The HIV-1 qPCR assay showing collective plots from replicate amplifications across the best 5 reverse transcriptases. The panels A, B and D show amplification tests with 10, 100 and 1000 Armored HIV RNA copies (panels A, B & C respectively) and their combined melting curves (panel D). Only Cq points above 25 were shown in the graphs to emphasize the differences among the enzymes assessed.
(0.65 MB EPS)

Figure S4 The average quantification cycle (Cq) obtained from seven RT enzymes that consistently amplified the 1,000 copies of input Armored HIV RNA standard. The straight (arRNA) were compared to spiked ones (FFPE+arRNA) to assess their reverse transcription success with known RNA from a typical FFPE tissue.
(0.21 MB EPS)
